# Effect of Employee Experience on Organizational Commitment: Case of South Korea

**DOI:** 10.3390/bs13070521

**Published:** 2023-06-21

**Authors:** Minkyung Lee, Boyoung Kim

**Affiliations:** Seoul Business School, aSSIST University, Seoul 03767, Republic of Korea; mk.lee@stud.assist.ac.kr

**Keywords:** employee experience, organizational commitment, job satisfaction, psychological well-being, mental toughness

## Abstract

This study’s purpose is to examine the effect of employee experience on job satisfaction, psychological well-being, and organizational commitment among corporate employees, with a specific focus on three factors: physical, technological, and cultural experiences. Given the growing importance of mental health management for employees, the study investigates the relationship between employee experience and mental toughness. A structural equation modeling research model was designed, and data were collected through a survey of 534 Korean employees. The analysis results show that cultural and physical experiences have a significant impact on organizational commitment, while technological experience does not have a significant impact. Furthermore, the study identifies that employee experience positively effects organizational commitment through job satisfaction and psychological well-being as mediators. It also reveals that the effect of employee experience on organizational commitment varies depending on the level of mental toughness. The findings suggest that managing employee experience can increase organizational commitment by improving job satisfaction and psychological well-being. Therefore, it is essential to be aware of individual mental toughness and its development. Hence this study highlights the importance of considering the physical, technological, and cultural experiences of employees in enhancing their well-being and commitment to the organization.

## 1. Introduction

As the business environment and organizational management continue to evolve, organizations are increasingly prioritizing the quality of work experience of their members over solely achieving organizational performance. In the past, the emphasis was on generating utility and productivity. However, in recent years, employee engagement has shifted towards employee experience, which focuses on creating a positive and engaging work environment that makes employees happy and fulfilled [1]. With the Fourth Industrial Revolution and the COVID-19 pandemic accelerating digital transformation, the new values and career perspectives of digital natives, particularly millennials, are driving the demand for change in business organizations [2,3,4,5,6]. Therefore, organizations are increasingly proposing various programs such as well-being, work–life balance, and workcations that consider the life cycle and values of organizational members, beyond narrow and temporary employee satisfaction or organizational culture improvement [7,8,9,10,11,12]. These programs are aimed at creating a positive work environment that fosters employee well-being, engagement, and commitment, which in turn leads to better organizational performance. In this context of change, the importance of employee experience management is particularly emphasized [13]. 

Employee experience refers to all interactions and touchpoints that employees experience in the organization [14,15]. This includes all aspects of the employee’s journey, from recruitment and onboarding to daily work tasks, performance management, and career development. It also refers to the overall perception of the company and the employee’s role resulting from complex interactions between the organization and its members [13]. For employees, employee experience becomes a meaningful everyday moment in the workplace [16], and it can have a significant impact on their job satisfaction, motivation, and overall well-being. While employee experience is often associated with the workplace, relationships with colleagues, compensation, and other tangible factors, it is, in essence, a more human experience that includes recognition and support, meaningful work, and individual ways of contributing to the organization [17]. 

As a result, the importance of managing employee experience has become a key aspect of modern organizational human resource management strategies [13]. To create a positive and engaging employee experience, organizations need to focus on building a culture of trust, fairness, and collaboration, where employees feel valued and supported [18]. This requires personalized and genuine experiences that cater to the individual needs and preferences of employees, as well as a commitment to ongoing improvement and innovation. To expand positive employee experiences, organizations can take various steps, such as building strong relationships with colleagues, promoting work–life balance, providing meaningful work opportunities, and having fair reward and management systems [19]. In a recent report, Gartner [20] emphasized that employee experience is the result of the cumulative effect of various factors such as business processes, systems, policies, work environment, department, leadership, and interactions with customers that employees perceive. According to their survey of 250 global companies, while 34% of the companies focused on increasing direct investment in individual employees such as incentives or welfare allowances, 64% of them prioritized improving the overall employee experience.

The quality of employee experience plays a significant role in determining organizational commitment and job satisfaction [13]. Previous studies have shown that employee experience has a positive impact on various aspects of organizational value, such as corporate innovation, productivity, customer satisfaction, profitability, and job performance, by influencing work engagement [21,22]. Furthermore, positive employee experience leads to organizational efficiency and affects engagement and positive job performance [15,18]. Positive employee experience also enhances job satisfaction [23] and contributes to individual psychological well-being within the organization, which in turn enhances concentration and job satisfaction [24].

Given the rapid changes in organizational culture and human resource management systems since the COVID-19 pandemic, the design of employee experiences that enhance job attitudes and activities is more important than ever. However, previous studies have treated employee experience as a similar concept or influence factor of organizational commitment [25,26]. Therefore, this study aims to empirically analyze the effect of employee experience, which consists of physical, technological, and cultural experiences, on job satisfaction, psychological well-being, and organizational commitment. Specifically, this paper identifies the factors among the three dimensions of employee experience that have a greater impact on organizational activities and to provide specific directions for future organizational HR management strategies. With the recent emphasis on stress management and mental health promotion among employees, this study investigates the effect of employee mental toughness on employee experience and job performance. Eventually, the importance of employee experience is validated in the changing organizational culture and management strategies and provides strategic implications for HR management practices that align with the current era.

## 2. Literature Review 

### 2.1. Concept of Employee Experience

Employee experience (EX) is a concept that can be compared to Customer Experience (CX), which represents the experience of customers, and User Experience (UX), which refers to the experience of users. It is the result of interactions that take place between employees and organizations and encompasses the overall perception of members of the organization towards their roles [13]. It is defined as a set of psychological and cognitive emotions related to the experiential benefits of employment and provides a positive, supportive, and personalized experience that enables all employees to contribute to the organization [17]. Moreover, employee experience is formed in the process of specific actions related to the job and the company, and this affects the attitudes and perceptions of employees. Plaskoff [27] emphasized that employee experience is characterized by continuous questioning, observation, and analysis of problem-solving processes and solutions from an employee perspective rather than an organizational perspective.

Recently, employee experience has become a factor that particularly makes young-generation employees proud of working for their company and want to make an impact on the world around them [28,29,30]. They want to know about the value the company creates, the problems it solves, and the purpose it serves. Many large organizations use technology such as corporate social networks to communicate their brand value to each employee and emphasize their role as agents in realizing it [31]. 

As shown in Table 1, while past employee experience was a part of specific programs for HR management, recent employee experience has evolved into programs or platforms that empower employees with decision making, participation in dynamic work environments, challenging project execution, diverse self-development opportunities, and permission to learn new ways to contribute [15,32].

According to Speicher and Francis [34], enhancing employee experience plays a vital role in reducing employee turnover and attracting top talent. Additionally, providing competitive employee experiences is effective in enhancing employee productivity or professionalism because it has more meaning than offering rewards or flexible working hours [35,36,37]. Mahadevan and Schmitz [38] and Laiho et al. [39] argued that an attractive employee experience provides opportunities for value creation in various areas such as process improvement, new product development, decision making, brand communication, employment, and training. However, to become a practical employee participation program rather than a uniform form, it needs to reconstruct business processes and personalize work experience in a meaningful way that caters to cognitive, emotional, and social needs. Therefore, to enhance employee experience, there is a growing trend towards monitoring employee needs, designing more engaging work routines, improving productivity and visibility, conducting internal experiments, and providing continuous support [14,18,40].

Morgan [1] defined employee experience as the intersection of employees’ expectations, demands, and needs and organizational design related to those expectations, demands, and needs, which results from the interaction between employees and the organization. Morgan proposed three dimensions of employee experience: physical, technological, and cultural experiences. Physical experience refers to the physical work environment where employees actually work, including workspace, facilities and equipment, lighting, noise, and ergonomics. Kamarulzaman et al. [41] found that office temperature, air quality, lighting, and noise conditions affect work concentration and productivity. Dul et al. [42] reported that physical work environment factors such as lighting and layout influence employee satisfaction. Furthermore, a comfortable and safe physical work environment is positively associated with job satisfaction and organizational commitment [43]. Technological experience refers to the resources and technology level that employees can use to perform their work effectively, including everything from applications to hardware, software, user interfaces, and design [44]. Chandwani et al. [45] and Moganadas and Goh [46] found that digital technology has a positive impact on employee experience management. Cultural experience refers to the values, beliefs, and norms of the organization that create the organizational atmosphere and everything that contributes to it, such as the quality of relationships between colleagues and managers, the degree of autonomy granted to employees, and the level of recognition and gratitude given to employees [47,48,49]. Tuisku and Houni [50] stated that sharing cultural experiences among employees can increase the organization’s social capital. Ultimately, these components of employee experience have a positive impact on employees’ job activities, motivation, active participation, and attitudes [51].

### 2.2. Employee Experience and Job Satisfaction

Job satisfaction refers to an individual’s emotional response to their role, environment, peer relationships, and various aspects of their job, and can be defined as a function of the extent to which an individual’s needs are met in the workplace [52,53]. Job satisfaction is an important component of organizational behavior because it has a significant impact on employee motivation, involvement, and performance [23,54]. Schwarzer and Hallum [55] expressed job satisfaction as the happiness or fulfillment that organizational members feel about their work, while Smircich [56] defined it as a holistic positive emotion relating to job-related desires, attitudes, values, beliefs, and the resulting positive attitude. Previous studies have shown that employee experience has a positive impact on employee engagement and influences the job satisfaction of organizational members [57,58,59,60].

The physical experience has been identified as an important factor influencing job satisfaction [61,62]. Ashraf et al. [63] stated that physical work experience affects job satisfaction and organizational performance. Aiken et al. [64] found that external factors such as collaboration spaces and amenity spaces influence employee satisfaction. Employees with greater freedom to choose their workspaces have higher job performance, innovation, and job satisfaction levels [65].

The technological experience, which involves the interaction of technology, structure, and organizational climate, has a significant impact on job satisfaction [66]. Employees who receive the necessary technical resources and training for their job perform better and have higher organizational commitment [67]. Organizations that invest in technology and innovative tools for their employees have higher employee satisfaction levels [68,69]. Additionally, information and communication technology (ICT) infrastructure, training, and support also have an impact on job satisfaction [70].

The cultural experience enhances employee productivity within a positive and healthy organizational culture [71]. A positive cultural experience promotes a sense of dedication to one’s work and connection to the organization, improves attitudes and job involvement, and enhances pride and loyalty as organizational members [59]. Autonomy in job roles, positive reinforcement, and meaningful feedback also contribute to a positive cultural experience and influence job satisfaction [72,73]. Based on these previous studies, the following hypotheses were designed for this study:

**Hypothesis** **1** **(H1).** *The physical experience of organizational members will have a positive (+) impact on job satisfaction.*

**Hypothesis** **2** **(H2).** *The technical experience of organizational members will have a positive (+) impact on job satisfaction.*

**Hypothesis** **3** **(H3).** *The cultural experience of organizational members will have a positive (+) impact on job satisfaction.*

### 2.3. Employee Experience and Psychological Well-Being

Psychological well-being refers to an individual’s mental and emotional health status, including factors such as positive emotions, personal growth, and a sense of meaning in life, which contribute to an overall sense of life satisfaction [74,75]. A life with high psychological well-being is characterized by a sense of control over the environment, a purpose in life, and motivation to realize one’s potential [75]. Individuals with high psychological well-being accept themselves as they are, maintain positive interpersonal relationships, and have the ability to regulate their behavior independently [76].

From the physical experience perspective, employees have a social identity in their workspace that affects their psychological well-being [24]. The degree to which individuals experience control over their job and work environment is associated with psychological ownership of their job and organization [77]. Vander Elst et al. [78] found that social support, participation, and job autonomy in remote work have a positive impact on employee well-being. Moen et al. [79] demonstrated that teams with flexible work arrangements experienced better job satisfaction, less burnout, and lower stress levels.

In terms of technological experience, digitalization has an impact on the mental health and psychological well-being of employees [80], and digital interventions in the workplace have a positive effect on psychological well-being [81]. Bordi et al. [82] investigated the relationship between digital communication in the workplace and well-being and found that the volume of digital communication, expectations of constant connectivity, quality of messages, adaptation of new tools, technical problems, and flexibility in communication all have an impact on well-being within the workplace.

Cultural experiences, such as organizational fairness, well-being, and feedback, also influence employees’ psychological factors. Organizational culture, including diversity, inclusivity, and fairness, affects job satisfaction and employee well-being [83]. Employee experience is a key factor that affects organizational innovation, productivity, job satisfaction, and talent acquisition and retention [84]. Sparr and Sonnentag [85] found that perceptions of the fairness of management feedback have a significant impact on employees’ psychological well-being, including job depression, anxiety, and turnover intentions. Furthermore, low organizational fairness leads to increased psychological distress [86]. Robbins et al. [87] demonstrated the relationship between perceptions of unfairness in the workplace and mental health. Based on these previous studies, the following hypotheses were formulated for this study:

**Hypothesis** **4** **(H4).** *The physical experience of organizational members will have a positive (+) impact on psychological well-being.*

**Hypothesis** **5** **(H5).** *The technical experience of organizational members will have a positive (+) impact on psychological well-being.*

**Hypothesis** **6** **(H6).** *The cultural experience of organizational members will have a positive (+) impact on psychological well-being.*

### 2.4. Employee Experience and Organizational Commitment

Organizational commitment is defined as the attitude of organizational members toward the organization they belong to, in terms of the degree to which employees identify with their organization’s goals, values, and culture, and the level of attachment they have [88,89,90,91]. It can also be described as the degree of willingness or attachment to stay in the organization [92], and the extent to which employees feel loyalty to the organization [93,94]. Organizational commitment of employees is considered an important factor that ultimately influences organizational performance, not only individual performance, but also turnover intentions, organizational silence, and employee engagement [95,96,97,98]. The core of employee commitment is the active and positive psychological state that is continually manifested in cognitive, emotional, and behavioral aspects related to work, in which members voluntarily draw out their inherent abilities for the organization’s benefit [99].

Yohn [100] explained that organizational commitment is the ultimate goal of organizational operations, and employee experience is the means to achieve that goal. Employee experience is a tool that ultimately leads to employee commitment and drives organizational purpose and change [25,101,102]. Saks [59] suggested through research that a positive employee experience in the organization leads to a higher level of organizational commitment. The philosophy of employee experience is recognized as an effective tool for achieving the highest level of employee commitment in the business environment [103], and Tucker [14] stated that most employers use employee experience as a strategy for employee commitment. In the end, a positive employee experience is considered a key to employee engagement and influences organizational efficiency and work performance [104].

In particular, Morgan [1] stated that physical, technological, and cultural employee experiences have a positive impact on employee commitment and ultimately on a company’s final values, such as corporate innovation, productivity improvement, customer satisfaction, corporate profit, and job performance. Hershatter and Epstein [30] explained that the younger generation, in particular, takes pride in the company they work for and wants to make an impact on the world around them, and therefore, their physical and cultural employee experiences within the organization can change their perception and affect their job efficiency and commitment. Based on these previous studies, the following hypotheses are proposed in this study.

**Hypothesis** **7** **(H7).** *The physical experience of organizational members will have a positive (+) impact on organizational commitment.*

**Hypothesis** **8** **(H8).** *The technical experience of organizational members will have a positive (+) impact on organizational commitment.*

**Hypothesis** **9** **(H9).** *The cultural experience of organizational members will have a positive (+) impact on organizational commitment.*

### 2.5. Job Satisfaction, Psychological Well-Being, and Organizational Commitment

Job satisfaction refers to the pleasure or contentment related to an individual’s job or work environment [105]. Job satisfaction is related to various organizational outcomes such as employee performance, turnover intention, and organizational commitment [106,107] and is linked to organizational commitment [108]. Although job satisfaction and organizational commitment are similar in terms of attitude, they are distinct in that job satisfaction pertains to attitudes towards objective job conditions, while organizational commitment pertains to a general attitude towards the organization [109]. Demir [110] revealed research results indicating that job satisfaction has a positive impact on the intention to remain in the organization and on organizational commitment. Gunlu, Aksarayli, and Şahin Perçin [111] reported that extrinsic job satisfaction and intrinsic job satisfaction significantly influence organizational commitment. Several previous studies have reported that job satisfaction has a positive impact on organizational commitment [112,113,114,115,116,117]. Aydogdu and Asikgil [118] proposed that job satisfaction has a positive impact on organizational commitment and that turnover intention has a negative relationship with job satisfaction and organizational commitment. Employees with high job satisfaction and organizational commitment are more likely to collaborate organically with colleagues, support organizational changes, and contribute to a positive organizational culture [108]. Based on these previous studies, the following hypothesis was designed:

**Hypothesis** **10** **(H10).** *The job satisfaction of organizational members will have a positive impact on organizational commitment.*

In Shuck and Reio’s [119] study, psychological well-being was found to be positively related to organizational commitment, with positive emotions and a sense of purpose having the strongest impact. Employees with high psychological well-being were found to have high job satisfaction and job involvement [120]. Meyer and Maltin [121] explained that psychological well-being and job satisfaction are positively related to organizational commitment. Brunetto et al. [122] and Garg and Rastogi [123] reported that psychological well-being has a positive and significant relationship with job involvement. Additionally, several previous studies have suggested that psychological well-being has a positive impact on job satisfaction, organizational commitment, and job performance [124,125]. Salimirad and Srimathi [126] emphasized that psychological well-being is a significant predictor of job satisfaction, organizational commitment, and job performance. Based on these previous studies, the following hypothesis was designed in this study.

**Hypothesis** **11** **(H11).** *The psychological well-being of organizational members will have a positive (+) impact on organizational commitment.*

### 2.6. Differences on the Level of Mental Toughness 

Mental toughness refers to the personality trait that describes an individual’s capacity to cope with stress, pressure, and adversity, and is composed of four sub-components: control, challenge, commitment, and confidence [127]. Reference [128] found that mental toughness is positively associated with job satisfaction. Yang and Hwang [129] suggested that job satisfaction is influenced by individual differences such as personality traits. Previous empirical studies have also demonstrated that mental toughness significantly affects organizational commitment and job satisfaction [130,131,132].

Moreover, Jones et al. [133] explained that employees use mental toughness as a resource to cope with work-related stress. According to Mojtahedi et al. [134], during the COVID-19 pandemic, individuals with higher levels of mental toughness showed relatively lower levels of negative emotions such as depression, stress, and anxiety caused by job loss. Mental toughness is positively related to life satisfaction [135], but is negatively related to attachment anxiety, avoidance, burnout, and other negative emotions [136]. Therefore, mental toughness can affect employees’ job satisfaction, psychological well-being, and organizational commitment, and higher levels of mental toughness may result in more positive outcomes. Based on previous research, this study hypothesizes the following:

**Hypothesis** **12** **(H12).** *The relationship between employee experience factors and job satisfaction, psychological well-being, and organizational commitment will differ depending on the level of mental toughness of employees.*

## 3. Research Methods

### 3.1. Research Model

**Figure 1:**
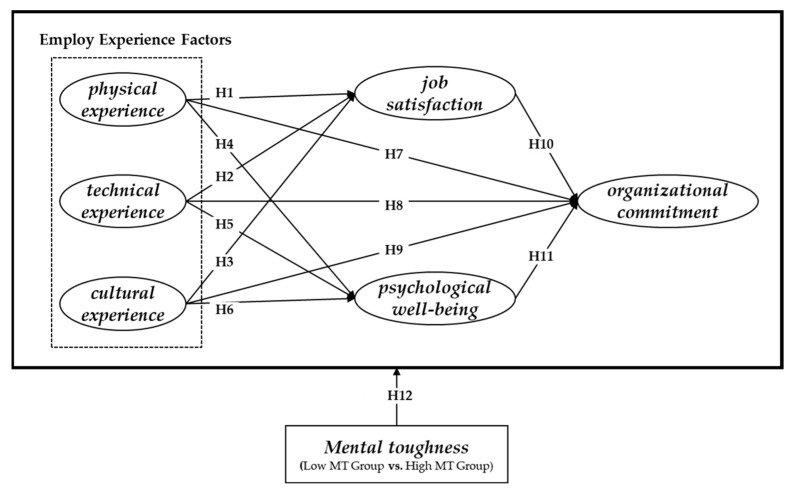
Research model.

Through the exploration of previous studies and theoretical discussions, this study aims to investigate the relationships between three factors of employee experience (physical, technical, and cultural), job satisfaction, psychological well-being, and organizational commitment. Additionally, we compare the differences based on individuals’ personality trait of mental toughness. To achieve this, we designed a research model, as shown in Figure 1, based on structural equation modeling to examine the impact of employee experience on organizational commitment and to investigate whether job satisfaction and psychological well-being mediate the relationship between employee experience and organizational commitment.

### 3.2. Measurement Variable and Data Collection

In order to collect data for analyzing this model, a survey was conducted. The survey questions were composed through prior research as shown in Table 2 below. The operational variables of the survey factors to be composed through the survey were also defined.

In this study, ‘employee experience’ refers to the overall journey that an employee experiences while interacting with the organization, based on the common ground between the employee’s expectations, needs, and hopes and those of the organization.

‘Job satisfaction’ refers to the emotions felt by an individual in various aspects of their job such as role acceptance, environment, and colleague relationships. It encompasses positive emotions such as needs, attitudes, values, and beliefs related to the job and the proactive attitude resulting from them. The variable was defined as a factor influencing job and work environment and long-term sustainability.

‘Psychological well-being’ refers to a high level of life characterized by an individual’s ability to choose and change their surroundings while maintaining positive interpersonal relationships and accepting themselves as they are based on a sense of well-being that means positive psychological functioning and experience. In this study, the variable was defined as an influential factor considered important for desirable life outcomes including achievements in work, education, and interpersonal relationships.

‘Organizational commitment’ refers to the degree of a firm mindset in which an organization member owns or wishes to devote their identity to the organization. In this study, the variable was defined as a factor influencing attitudes towards the organization and attachment and commitment to goals, loyalty, and degree of feeling.

In addition, ‘mental toughness’ is a personality trait that determines how an individual behaves in stressful or challenging situations such as opportunities and challenges. In an organizational environment, there are many situational variables such as stress or challenge that can have a significant impact on mental toughness.

As shown in Table 2 below, these defined variables were composed of a total of 31 survey questions in the questionnaire. The employee experience scale was composed of 10 questions based on Morgan’s [1] prior research to fit the direction of this study. Job satisfaction was composed of 4 questions based on Brayfield and Rothe’s [137] and Thompson and Phua’s [138] prior research. Psychological well-being was composed of 5 questions based on Ryff’s [73] and Burns and Machin’s [139] prior research to fit the direction of this study. Organizational commitment was composed of 4 questions on emotional commitment based on Meyer and Allen’s [88] and Lee et al.’s [140] prior research. The mental toughness scale used 8 items from the Mental Toughness Questionnaire (MTQ4Cs) developed by Clough and Strycharczyk [141].

For data analysis, SPSS 27.0 was used to analyze demographic characteristics, descriptive statistics, exploratory factor analysis, etc. For path analysis of hypotheses, AMOS 27.0 was used to conduct confirmatory factor analysis based on structural equation modeling, model verification, and path analysis.

**Table 2:** Variable definitions and measurement items.

Factors	Measurement Items	References
Physical Experience	- Our company provides various workspaces such as conference rooms, collaboration spaces, open spaces, and cafes. - I am proud to bring guests such as friends or family to the office. - Our company provides and encourages a free and flexible work environment.	Morgan [1]
Technical Experience	- Our company’s systems are easy to use and useful. - The company supports all employees in using the company’s technology/systems. - Our company’s technology/operating systems are continuously improved by incorporating employee feedback.	
Cultural Experience	- I feel that I am treated fairly at the company. - Our company encourages diversity and inclusion. - Our company is concerned with the physical and mental well-being of its employees. - Our company provides opportunities and resources for employee growth.	
Job Satisfaction	- I am satisfied with what I am currently doing at the company. - I’m enjoying my current job. - I feel rewarded for what I do now. - I want to continue what I do now.	Brayfield and Rothe [137], Thompson and Phua [138]
Psychological Wellbeing	- I feel happy when I compare myself to friends and relatives. - I generally manage my personal or financial problems well. - Looking back on my life, I am satisfied with the current results.	Ryff [73], Burns and Machin [139]
Organizational Commitment	- I have a high sense of belonging to the company. - I am attached to my department or company. - Working in this organization is personally meaningful. - I feel that the problem with my department or company is my problem.	Meyer and Allen [88], Lee et al. [142]
Mental toughness	- I am generally confident in my own abilities - I don’t mind setbacks, there’s always something to learn from them - I can generally be relied upon to complete the tasks I am given - Sometimes I just can’t hold my emotions inside (R) - I do not usually criticise myself even when things go wrong - I usually enjoy a challenge - Challenges usually bring out the best in me - If I feel somebody is wrong, I am not afraid to argue with them	Clough and Strycharczyk [143]

### 3.3. Demographic Information of the Data

In this study, an online survey was conducted through random sampling targeting employees working in Korean companies for the survey. The survey was conducted over a period of 4 weeks from 15 December 2022, using a self-report questionnaire. A total of 613 surveys were collected and 534 surveys were used for analysis after excluding 79 surveys that were insincerely responded to. Detection of insincere responses was determined based on one-line responses and psychological measurement consistency among non-interventional post hoc detection methods. A one-line response (long string) is when the same response is given to 9 consecutive questions out of 33 questions, and lack of consistency in agreement/disagreement is when the correlation between individual responses between pairs of questions related to agreement/disagreement is less than 0.3 [138,139].

As shown in Table 3, the gender ratio of the survey participants was evenly composed, with 49.8% male and 50.2% female. The age distribution was 20.8% in their 20s, 30.3% in their 30s, 25.7% in their 40s, and 23.2% in their 50s, with a symmetrical distribution between those in their 20–30s and those in their 40–50s. The educational background was distributed as follows: below college graduation at 10.7%, vocational college graduation at 15.2%, university graduation at 60.1%, and master’s/doctorate at 14%. The workplace sizes were composed of 34.3% with less than 50 employees, 32.8% with 50 to less than 300 employees, 14.2% with 300 to less than 1000 employees, and 18.7% with more than 1000 employees. The industries were manufacturing at 28.5%, service at 19.3%, construction/rental/distribution at 17.0%, professional group at 24.5%, and others at 10.7%. The jobs were office/administration at 62.2%, sales/marketing at 12.7%, research/development at 9.9%, production/technology at 9.9%, and others at 5.2%, with a high distribution of office/administration jobs. The job positions were contract (non-regular employees) at 9.4%, team member at 51.5%, middle manager at 33.0%, and senior manager at 6.2%, showing a higher distribution of managers or higher compared to general companies.

## 4. Results

### 4.1. Results of Reliability and Validity Analysis

The analysis results of the reliability and convergent validity of the measurement model, as shown in Table 4, were all found to be satisfactory. The factor loading ranged from 0.622 to 0.854, indicating satisfactory levels of convergent validity. The composite reliability values were between 0.779 and 0.891, indicating acceptable levels of internal consistency. The *t*-values were greater than 6.5, confirming statistical significance. The average variance extracted (AVE) values ranged from 0.540 to 0.667, and the Cronbach’s alpha values ranged from 0.813 to 0.864, thus ensuring convergent validity.

An unrotated factor analysis was conducted using Harman’s single-factor test method [144] with six variables for the independent, mediating, and dependent variables. As a result, six factors with explanatory power (eigenvalue > 1.00) were extracted and their cumulative variance value accounted for 67.817%. The variance value of the factor with the greatest explanatory power was 35.182%, and the remaining five factors accounted for 32.635% of the total variance, confirming that there was no general factor causing common method bias.

The analysis of the fit of the structural equation measurement model showed that χ^2^(df) was 360.8 and χ^2^/df was 1.694. The goodness-of-fit index (GFI) was 0.945, the adjusted goodness-of-fit index (AGFI) was 0.929, the normal fit index (NFI) was 0.941, and the root mean square error of approximation (RMSEA) was 0.036, indicating that the model fit indices were statistically significant.

The analysis of AVE values and correlation coefficients among latent variables in this study showed, as presented in Table 5, that the square root of AVE for each latent variable was larger than the correlation coefficients between latent variables, indicating discriminant validity.

### 4.2. Results of Structural Equation Model Analysis

The fitness of the structural model was examined and the results indicated that the χ^2^(p) was 437.102 and the χ^2^/df was 2.024, as presented in Table 6. The Goodness-of-Fit-Index (GFI) and Normal Fit Index (NFI) were both greater than 0.9, with values of 0.935 and 0.928, respectively. The value of Root Mean Square residual (RMS) was 0.035, Adjusted Goodness-of-Fit-Index (AGFI) was 0.917, and Root Mean Square Error of Approximation (RMSEA) was 0.044, indicating that the fitness constructs were excellent and that the model fit was significant. The Comparative Fit Index (CFI), which represents the explanatory power of the model, was not influenced by the sample and it showed a value of 0.962, and the Tucker–Lewis Index (TLI), which judges the explanatory power of the structural model, was 0.955, indicating that the basic model was very suitable.

Through path analysis of the structural equation model, the hypothesis that employee experience affects organizational commitment through job satisfaction and psychological well-being was examined, as presented in Table 6. Physical experience had a positive effect (2.979, *p* < 0.05) on job satisfaction with a path coefficient of 0.182. However, the effect of technical experience on job satisfaction was rejected. The path coefficient for the cultural experience on job satisfaction was 0.330, indicating a positive effect (4.621, *p* < 0.001), and was accepted.

Next, physical experience had a positive effect (4.273, *p* < 0.001) on psychological well-being with a path coefficient of 0.307, and the hypothesis was accepted. Technical experience was also accepted with a positive effect (3.237, *p* < 0.01) on psychological well-being with a path coefficient of 0.028. However, the effect of cultural experience on psychological well-being was rejected.

The path coefficient for physical experience on organizational commitment was 0.170, indicating a positive effect (3.071, *p* < 0.01), and it was accepted. The path coefficient for cultural experience on organizational commitment was 0.192, indicating a positive effect (3.299, *p* < 0.001), and was also accepted. On the other hand, the effect of technical experience on organizational commitment was rejected. The path coefficient for job satisfaction on organizational commitment was 0.451, indicating a positive effect (8.929, *p* < 0.001), and it was accepted. Lastly, psychological well-being had a positive effect (6.964, *p* < 0.001) on organizational commitment with a path coefficient of 0.321, and the hypothesis was accepted.

### 4.3. Results of the Direct, Indirect, and Total Effects Analysis

As shown in Table 7, the results of analyzing the direct, indirect, and total effects between the variables of the model showed that the sub-factors of employee experience, physical experience, and cultural experience had significant direct and indirect effects on organizational commitment through job satisfaction as a mediator, resulting in a significant total effect. However, the effect of technical experience on organizational commitment through job satisfaction as a mediator was not significant. In addition, physical experience and cultural experience had significant direct and indirect effects on organizational commitment through psychological well-being as a mediator, resulting in a significant total effect. However, the effect of technical experience on organizational commitment through psychological well-being as a mediator was not significant.

These findings suggest that physical and cultural experiences can increase organizational commitment by enhancing job satisfaction and psychological well-being. Examining the mediating effects between exogenous and endogenous variables, job satisfaction acted as a partial mediator between physical experience and organizational commitment as well as between cultural experience and organizational commitment. Similarly, psychological well-being acted as a partial mediator between physical experience and organizational commitment and also acted as a partial mediator in the relationship between cultural experience and organizational commitment.

### 4.4. Moderating Effect

In order to verify hypothesis 12 that the effect of employee experience on organizational commitment through job satisfaction and psychological well-being as mediators will vary depending on the level of mental toughness, a personal characteristic, a multi-group analysis was conducted. First, in order to categorize mental toughness, the average (m = 3.46) of the items measuring mental toughness was calculated, and respondents who were evaluated above the average were classified as a high-mental-toughness group (n = 267) and those who were evaluated below the average were classified as a low-mental-toughness group (n = 267).

Before testing the mediation effect, we confirmed whether the measurement instruments were perceived equally between the two groups through measurement invariance testing. Measurement invariance testing examines whether there are differences in the distribution of factor loadings between a constrained model, which constrains factor loadings to be equal across groups using confirmatory factor analysis, and an unconstrained model without such constraints. The test results should yield a *p*-value greater than 0.05 to ensure measurement invariance. The measurement invariance test results for the high-mental-toughness group and the low-mental-toughness group are presented in Table 8. The difference in degrees of freedom between the constrained model and the unconstrained model is 17. The change in chi-square (Δχ^2^) is 26.906, and the *p*-value (0.059) indicates that the difference is not statistically significant. Thus, it was found that the high-mental-toughness group and the low-mental-toughness group perceive the measurement variables equally.

The interpretation of the moderation effects analysis results for the two groups of mental toughness, as presented in Table 9, is as follows.

First, in the effect of employee experience on job satisfaction, physical experience showed no significant difference between the low-mental-toughness group (β = 0.055, *p* > 0.05) and the high-mental-toughness group (β = 0.093, *p* > 0.05), and technical experience also showed no significant difference between the low-mental-toughness group (β = −0.007, *p* > 0.05) and the high-mental-toughness group (β = 0.072, *p* > 0.05). On the other hand, cultural experience was significant in both the low-mental-toughness group (β = 0.247, *p* < 0.05) and the high-mental-toughness group (β = 0.495, *p* < 0.001), indicating that there was a difference depending on mental toughness.

Second, in the effect of employee experience on psychological well-being, physical experience had a significant effect in the high-mental-toughness group (β = 0.193, *p* < 0.05) but not in the low-mental-toughness group (β = 0.147, *p* > 0.05). Therefore, there is a difference in the influence of physical experience depending on mental toughness and it can be interpreted that positive physical experiences increase psychological well-being only for those with high mental toughness. Technical experience was not significant in either the low-mental-toughness group (β = 0.190, *p* > 0.05) or the high-mental-toughness group (β = 0.135, *p* > 0.05), indicating that there was no difference depending on mental toughness. Cultural experience was also not significant in either the low-mental-toughness group (β = −0.193, *p* > 0.05) or the high-mental-toughness group (β = 0.176, *p* > 0.05), indicating that there was no difference depending on mental toughness.

Third, in the effect of employee experience on organizational commitment, physical experience had a significant effect in the high-mental-toughness group (β = 0.165, *p* < 0.05) but not in the low-mental-toughness group (β = 0.158, *p* > 0.05). Therefore, there is a difference in the influence of physical experience depending on mental toughness and it can be interpreted that positive physical experiences increase organizational commitment only for those with high mental toughness. Technical experience was not significant in either the low-mental-toughness group (β = −0.0048, *p* > 0.05) or the high-mental-toughness group (β = 0.015, *p* > 0.05), indicating that there was no difference depending on mental toughness. Cultural experience was significant in both the low-mental-toughness group (β = 0.238, *p* < 0.05) and the high-mental-toughness group (β = 0.239, *p* < 0.05), indicating that there was a difference depending on mental toughness. The effect of psychological well-being on organizational commitment was significant in both the high-mental-toughness group (β = 0.213, *p* < 0.01) and the low-mental-toughness group (β = 0.302, *p* < 0.001), with greater influence of psychological well-being in the low-mental-toughness group.

## 5. Conclusions

### 5.1. Findings and Discussions

This study empirically analyzed the relationship between job satisfaction, psychological well-being, and organizational commitment with the three factors that make up employee experience in a corporate organization: physical, technical, and cultural experience as independent variables. In addition, the relationship between employee experience factors and organizational commitment through job satisfaction and psychological well-being was analyzed. The results of this analysis were derived to determine how these relationships differ depending on the level of mental toughness of organizational members. In this study, the following major research findings were discovered.

First, it was confirmed that positive employee experiences in physical and cultural environments increase organizational commitment. This research finding implies that positive employee experience is an important variable that affects job satisfaction, consistent with previous studies [59,60,145]. Analysis results showed that the cultural aspect had the greatest impact, followed by the physical environment, and the technological dimension did not have a significant impact. This means that new systems or platforms that lead to technical experiences in a digital management environment can stimulate organizational members as new employee experiences but ultimately do not affect their job attitudes or behaviors. Ultimately, it clearly shows that cultural environmental factors such as a positive atmosphere, climate in the organization, fairness and diversity, and opportunities for growth can be effective in stimulating positive employee experiences.

Second, it was confirmed that employee experience has an impact on job satisfaction and psychological well-being, which in turn has a positive impact on organizational commitment. Ultimately, positive employee experience is an important factor in increasing job satisfaction, reducing stress, and promoting psychological well-being to induce stable job engagement [146]. Particularly, regarding psychological well-being, all three dimensions of physical, technological, and cultural experiences had a significant impact and affected job engagement. This can be interpreted as meaning that all activities that affect organizational members during their work process have a high correlation with psychological factors. In conclusion, it is essential to consider the psychological well-being of organizational members to enhance work productivity and efficiency, as well as to improve job satisfaction and organizational commitment. Moreover, the approach to strengthening physical, technical, and cultural experience factors that can enhance psychological well-being can also be considered.

Third, this study illuminated the relationship between employee experience and mental toughness. The research results showed that mental toughness, a personal characteristic trait, affects employee experience and job satisfaction, psychological well-being, and organizational commitment. The physical and cultural experiences had different effects on organizational commitment depending on individual mental toughness levels. In particular, physical experience had a positive effect on organizational commitment only for those with high mental toughness [147]. This suggests that even if the same physical and cultural experiences are given in an organization, the effect on employee commitment may vary depending on individual personality traits. Therefore, improving mental toughness can directly affect employee experience beyond simply being a personal characteristic factor of organizational members and ultimately affect job satisfaction and organizational commitment.

### 5.2. Research Implications

This study highlights the importance of employee experience, which has gained significant attention in recent practice, along with employee productivity and engagement. The study empirically analyzed the influence of job satisfaction, psychological well-being, and organizational commitment on employee experience. Based on these research findings, several practical implications can be suggested.

First, companies need to create a strong employee experience to enhance employee retention, skills development, knowledge transfer, and business improvement. Although the importance of key talent retention for corporate growth is emphasized, the level of job engagement among millennials is decreasing. Therefore, it is essential to create a strong employee experience to attract and retain top talent, enhance employee skills, and increase organizational loyalty and trust. By creating a positive employee experience and fostering organizational culture, employees can voluntarily and actively contribute to improving productivity and efficiency in human resource management. Furthermore, employee experience can also affect the formation of employees’ work ethic. The positive employee experience in the organization system is a part of their life under capitalism, which can not only strengthen organizational performance or job competency, but also promote employees’ work ethics.

Second, it is necessary to consider maximizing physical and cultural experience factors in enhancing employee experience. With the emergence of the MZ generation, work–life balance, flexible working environment, diverse workspaces, flexible technology/systems, fairness and diversity, growth, and happiness are emerging as important management factors. Therefore, companies can design organizations where employees want to come to work by focusing on physical and cultural dimensions that fit into the current environment. Additionally, because the COVID-19 pandemic has strengthened flexible work arrangements, significant changes in physical experience factors are required. When considering promoting work in a virtual space centered on a smart office, it is necessary to expand positive physical experiences and maximize diversity in cultural experience aspects to enhance job satisfaction and engagement.

Third, it is essential to consider a multidimensional integration strategy for cultural experiences in influencing organizational commitment. Technical experience for systems or processes for work has limitations in leading to organizational commitment. However, considering the characteristics of organizational members who are familiar with various digital platforms and social media, various personnel management programs that maximize employee experience based on digital technology experience need to be considered. Moreover, by designing customized employee experiences that consider employees’ personalities and individual characteristics through advanced HR management systems based on big data or people analytics, it is possible to establish HR management strategies that motivate employees and maximize their job engagement.

### 5.3. Research Limitations and Future Plans

Despite the significance and implications of this research, this study has the following limitations. First, this study is a study targeting employees of Korean companies and has limitations in generalizing research results. In future research, it will be necessary to conduct research targeting organizational members from various countries. Furthermore, it should continue to refine and specify research on employee experience through comparative analysis by industry type, region type, or country type.

Second, this study utilized three factors of physical, technological, and cultural experiences, based on previous studies, as the constituting elements of employee experience. In future research, the utilization of a qualitative research methodology can be employed to redefine the fundamental factors comprising employee experience, while also considering the expandability of research that accurately reflects the work environment, resignation, or changing jobs. Additionally, investigating the impact of employee experiences, such as alienation, exploitation, emotions, or potential risk of burnout, in relation to mental toughness would prove advantageous.

Third, splitting continuous variables into discrete groups is known to have poor implications for statistical power. The moderation models for latent variables are not well developed, so splitting the variable into groups probably occurred in this research. Nevertheless, it would be a limitation of the work. Statistical measurement methods for more specific and detailed classification methods should be discussed in measuring mental strength as a continuous variable.

## Figures and Tables

**Table 1:** Changes in employee experience.

Old Rules	New Rules
Employee experience defined by annual engagement surveys	Employee experience defined as a holistic view of life at work, requiring constant feedback, action, and monitoring
Culture is a topic on the company website and perhaps on the wall, but not measured or defined through behavior	Company uses tools and behaviors to measure, align, and improve culture during change, M&A, and other major initiatives
Companies have a series of HR leaders across recruiting, learning, rewards, engagement, and other HR service	Companies have someone responsible for the complete employee experience, focused on employees’ journeys, experiences, engagement, and culture
Compensation, benefits, and rewards are managed with a focus on benchmarking and fairness	Compensation, benefits, rewards, and recognition designed to make people’s life better and balance financial and nonfinancial benefits
Wellness and health programs are focused on safety and managing insurance costs	Companies have an integrated program for employee well-being, focused on the employee, her family, and her entire experience at life and work
Rewards are designed to cover salary, overtime, bonus, benefits, and stock options	Rewards also include nonfinancial rewards: meals, leaves, vacation policy fitness, and wellness program
Employee self-service is viewed as a technology platform that makes it easy to complete HR transactions and reports	The employee experience platform is designed, mobile, and includes digital apps, prescriptive solutions based on employee journeys, and ongoing communications that support and inspire employees

Reference: Deloitte University Press. 2017 [33].

**Table 3:** Demographic information of survey participants.

Section		Frequency	Ratio (%)
Gender	Male	266	49.8%
	Female	268	50.2%
Age	20~29	111	20.8%
	30~39	162	30.3%
	40~49	137	25.7%
	50s and above	124	23.2%
Education	High School	57	10.7%
	Junior College	81	15.2%
	University	321	60.1%
	Master’s and Doctorate	75	14%
Workplace Size	Below 50 employees	183	34.3%
	50–300 employees	175	32.8%
	300–1000 employees	76	14.2%
	above 1000	100	18.7%
Business Industry	Manufacturing industry	152	28.5%
	Service industry	103	19.3%
	Construction/lease/distribution	91	17.0%
	Professional group	131	24.5%
	others	57	10.7%
Job Type	Office/management	332	62.2%
	Sales/Marketing	68	12.7%
	Research/development	53	9.9%
	Production/Technology	53	9.9%
	others	28	5.2%
Job position	Contract (non-regular employees)	50	9.4%
	Team Member	275	51.5%
	Middle Manager	176	33.0%
	Senior Manager	33	6.2%

**Table 4:** Results of reliability and convergent validity test.

Variables	Measurement Questions	Standard Loading	Standard Error	*t* Value (p)	CR	AVE	Cronbach α
Physical Experience	PE1	0.784			0.779	0.540	0.813
	PE2	0.767	0.059	16.527 ***			
	PE3	0.758	0.061	16.436 ***			
Technical Experience	TE1	0.787			0.846	0.647	0.833
	TE2	0.853	0.057	18.447 ***			
	TE3	0.726	0.054	16.477 ***			
Cultural Experience	CE1	0.777			0.870	0.627	0.864
	CE2	0.785	0.054	18.569 ***			
	CE3	0.854	0.052	20.279 ***			
	CE4	0.720	0.052	16.844 ***			
Job Satisfaction	JS1	0.754			0.889	0.667	0.850
	JS2	0.763	0.061	17.027 ***			
	JS3	0.811	0.057	18.046 ***			
	JS4	0.738	0.054	16.456 ***			
Psychological Well-being	PWB1	0.685			0.891	0.621	0.827
	PWB2	0.658	0.065	13.196 ***			
	PWB3	0.668	0.068	13.367 ***			
	PWB4	0.715	0.060	14.167 ***			
	PWB5	0.777	0.067	15.107 ***			
Organizational Commitment	OC1	0.622			0.863	0.614	0.819
	OC2	0.731	0.085	13.529 ***			
	OC3	0.816	0.086	14.534 ***			
	OC4	0.761	0.079	13.898 ***			

Measurement model fit: χ^2^(df) 360.8, χ^2^/degree of freedom 1.694, RMS 0.033, GFI 0.945, AGFI 0.929, NFI 0.941, TLI 0.97, CFI 0.975, RMSEA 0.036. Note: *** *p* < 0.001.

**Table 5:** Correlation matrix and AVE.

	AVE	PE	TE	CE	JS	PWB	OC
Physical Experience	0.540	**0.735**					
Technical Experience	0.647	0.439 ***	**0.804**				
Cultural Experience	0.627	0.621 ***	0.581 ***	**0.792**			
Job Satisfaction	0.667	0.426 ***	0.509 ***	0.387 ***	**0.817**		
Psychological Well-being	0.621	0.395 ***	0.303 ***	0.346 ***	0.491 ***	**0.788**	
Organizational Commitment	0.614	0.586 ***	0.603 ***	0.448 ***	0.732 ***	0.629 ***	**0.784**

Note: *** *p* < 0.001/The square root of AVE is shown in bold letters.

**Table 6:** Results of hypothesis test.

	Hypothesis (Path)	SRW ^1^	*t* Value	Support
H1	Physical Experience → Job Satisfaction	0.182	2.979 *	accepted
H2	Technical Experience → Job Satisfaction	0.115	1.944	rejected
H3	Cultural Experience → Job Satisfaction	0.330	4.621 ***	accepted
H4	Physical Experience → Psychological Well-being	0.307	4.273 ***	accepted
H5	Technical Experience → Psychological Well-being	0.208	3.237 **	accepted
H6	Cultural Experience → Psychological Well-being	0.007	0.088	rejected
H7	Physical Experience → Organizational Commitment	0.170	3.071 *	accepted
H8	Technical Experience → Organizational Commitment	−0.020	−4.120	rejected
H9	Cultural Experience → Organizational Commitment	0.192	3.299 ***	accepted
H10	Job Satisfaction → Organizational Commitment	0.451	8.929 ***	accepted
H11	Physical Experience → Organizational Commitment	0.321	6.964 ***	accepted

Structural model fit: χ^2^(df) 437.102, χ^2^/degree of freedom 2.024, RMS 0.035, GFI 0.935, AGFI 0.917, NFI 0.928, TLI 0.955, CFI 0.962, RMSEA 0.044. ^1^ Standardized regression weights. Note: * *p* < 0.05, ** *p* < 0.01, *** *p* < 0.001.

**Table 7:** Total effect, direct effect, and indirect effect.

Hypothesis (Path)	Direct Effect	Indirect Effect	Total Effect
PE → JS → OC	0.170 *	0.181 **	0.351 **
TE → JS → OC	−0.020	0.119	0.099
CE → JS → OC	0.192 ***	0.151 **	0.343 **
PE → PWB → OC	0.170 *	0.181 **	0.351 **
TE → PWB → OC	−0.020	0.119	0.099
CE → PWB → OC	0.192 ***	0.151 **	0.343 **
PE → OC	0.170 *		0.170 *
TE → OC	−0.020		−0.020
CE → OC	0.192 ***		0.192 ***
JS → OC	0.451 ***		0.451 ***
PWB → OC	0.321 ***		0.321 ***

Note: * *p* < 0.05, ** *p* < 0.01, *** *p* < 0.001.

**Table 8:** Evaluation results of significant differences between the groups.

Model	DF	CMIN	P	NFI Delta-1	IFI Delta-2	RFI rho-1	TLI rho2
Unconstrained model	17	26.906	0.059	0.005	0.005	0.000	0.000

**Table 9:** Total Moderating Effect of Mental Toughness.

Paths		Low-MT Group (n = 267)		High-MT Group (n = 267)	
		Estimate (β)	*t*-Value	Estimate (β)	*t*-Value
H12	PE → JS	0.055	0.535	0.093	1.151
	TE → JS	−0.007	−0.079	0.072	0.835
	CE → JS	0.247	2.324 *	0.495	4.846 ***
	PE → PWB	0.147	1.392	0.193	2.040 *
	TE → PWB	0.190	2.064	0.135	1.358
	CE → PWB	−0.193	−1.781	0.176	1.585
	PE → OC	0.158	1.725	0.165	2.214 *
	TE → OC	−0.048	−0.613	0.015	0.193
	CE → OC	0.238	2.444 *	0.239	2.466 *
	JS → OC	0.491	5.549 ***	0.403	5.180 ***
	PWB → OC	0.302	3.800 ***	0.213	3.153 **

Note: * *p* < 0.05, ** *p* < 0.01, *** *p* < 0.001.

## Data Availability

Not applicable.
